# Supplementary figures and images for: Proteasome-Associated Proteins, PA200 and ECPAS, Are Essential for Murine Spermatogenesis
Source: Biomolecules. 2023 Mar 24;13(4):586. doi: 10.3390/biom13040586 (PMC10135743; doi:10.3390/biom13040586)

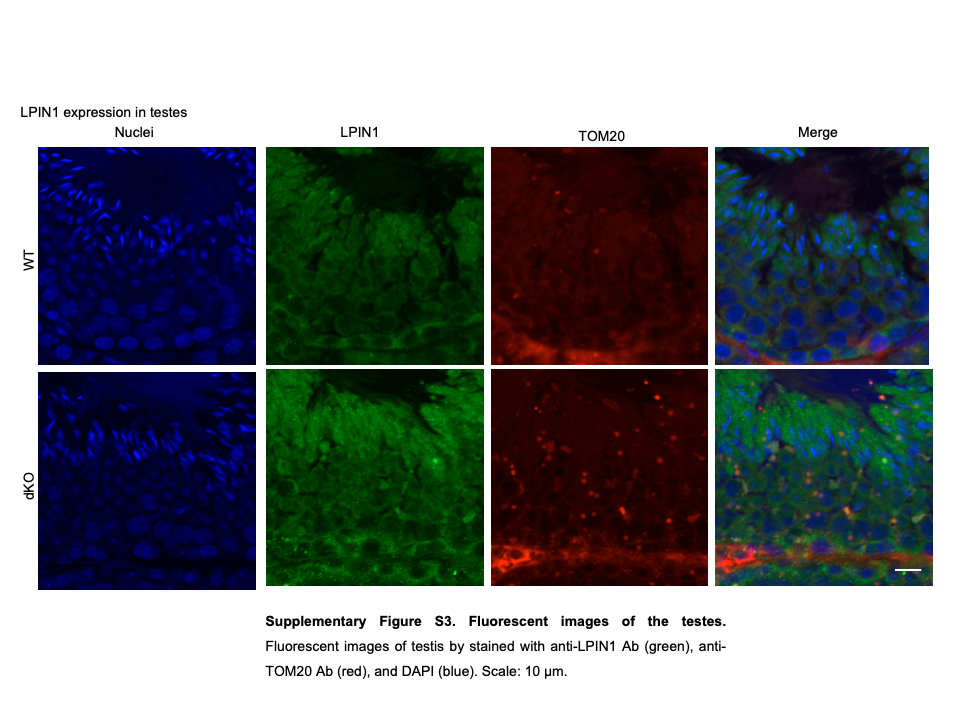

Supplement: Supplementary file 1 [file biomolecules-13-00586-s001.zip › supplementary files/Figure S3.tiff]

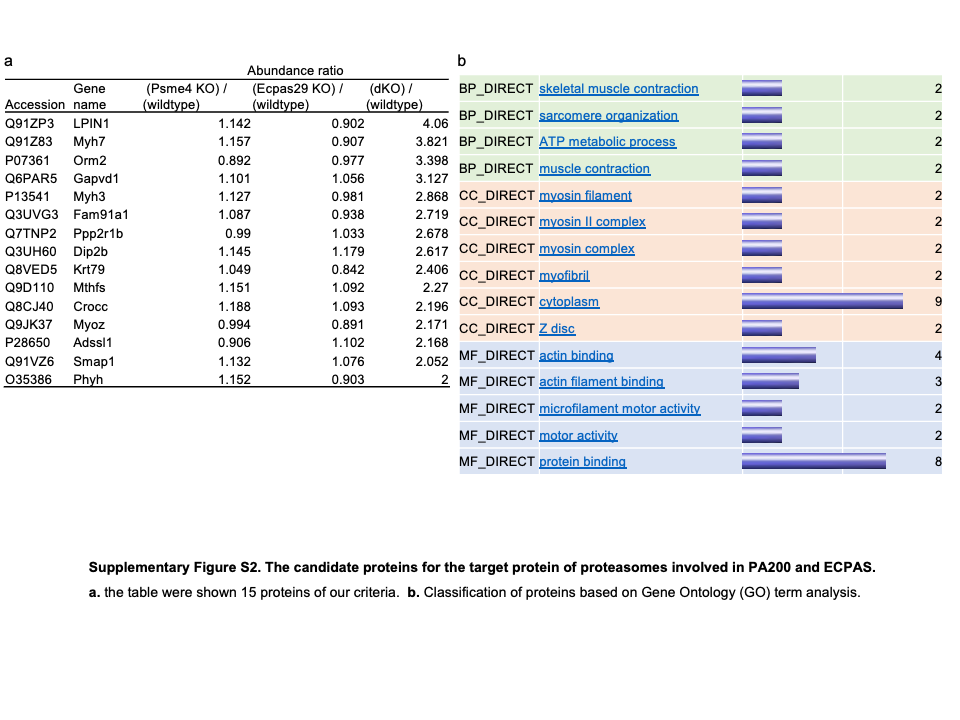

Supplement: Supplementary file 1 [file biomolecules-13-00586-s001.zip › supplementary files/Figure S2.tiff]

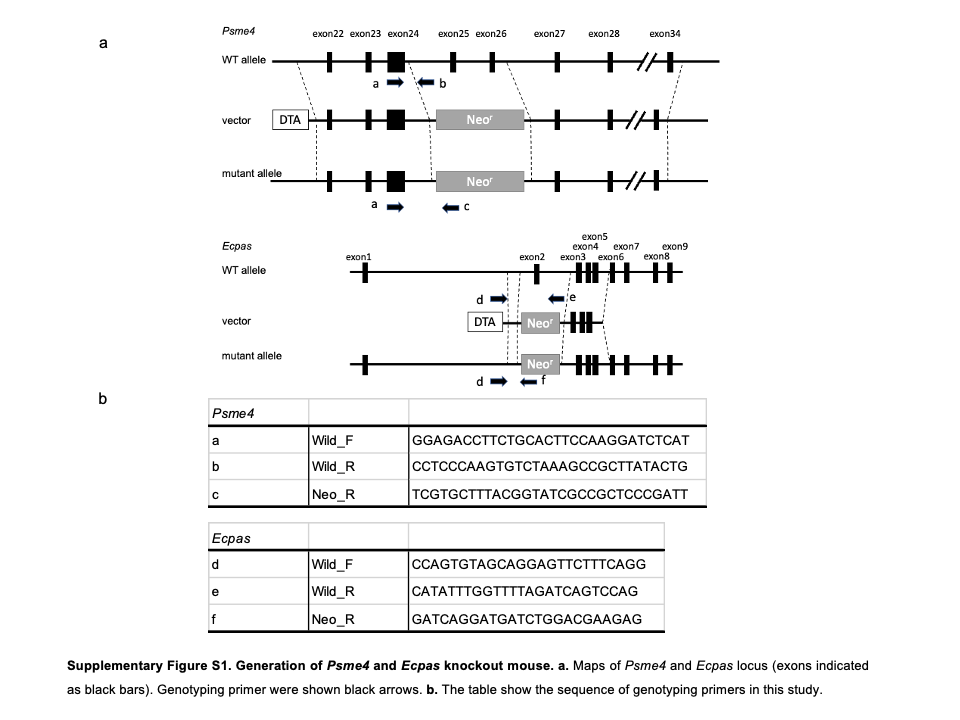

Supplement: Supplementary file 1 [file biomolecules-13-00586-s001.zip › supplementary files/Figure S1.tiff]
